# Surgeon's Impact on Opioid Epidemic Following Uncomplicated Laparoscopic Appendectomy and Cholecystectomy

**DOI:** 10.7759/cureus.25160

**Published:** 2022-05-20

**Authors:** Aakash Trivedi, James Yang, Daniel Barbash, Felippe Sartorato, Daniel J Scheinberg, Marc Meyers, Jamshed Zuberi, Benjamin Rebein

**Affiliations:** 1 Surgery, Saint Joseph's University Medical Center, Paterson, USA; 2 General Surgery, Saint Joseph's University Medical Center, Paterson, USA; 3 Anesthesiology, Saint Joseph's University Medical Center, Paterson, USA; 4 Trauma and Acute Care Surgery, Saint Joseph's University Medical Center, Paterson, USA

**Keywords:** cholecystectomy, appendectomy, narcotics, laparoscopic, opioid

## Abstract

The opioid crisis in the United States remains a major issue that is directly linked to the prescribing practices of physicians. There is a lack of consistency in post-operative prescribing of narcotic medications. We have designed a retrospective study to evaluate factors that contribute to the prescription of opioids following common laparoscopic procedures. In this study, we analyzed peri-operative medications and pain requirements and how they relate to the frequency in which narcotics are prescribed at Saint Joseph’s University Medical Center (SJUMC), a level two trauma center and teaching hospital. We also studied how the frequency of narcotic prescriptions is related to patient demographics and surgeon practices. We propose that standardizing pain medication protocols will be an effective way to decrease overall narcotic use as well as prescriptions for common laparoscopic procedures.

## Introduction

Opioids are a very common medication used to control acute and chronic pain; however, the epidemic of opioid addiction, abuse, and overuse has become a salient topic in the contemporary medical literature. This heightened focus comes in response to an astoundingly morbid series of epidemiologic studies exposing trends. One hundred and thirty Americans die every day from opioid overdose [1], and one out of 16 opioid naïve patients exposed to opioids after surgery will become persistent users [2]. Surgeons have been identified as one of the causes of overprescribing opioids in the United States [3]. Unused prescriptions contribute to the opioid epidemic. Centers for Disease Control (CDC) have provided clear updates on limiting opioids prescribed for various types of chronic pain; however, there has been less guidance on how to manage acute pain for common laparoscopic surgical procedures [4,5].

It is imperative to identify current peri-operative pain management strategies to develop more effective post-operative pain control and lower narcotic prescriptions. We reviewed all patients from May 1, 2017, to April 30, 2018, who underwent uncomplicated laparoscopic appendectomies and laparoscopic cholecystectomies in an effort to identify surgeons’ opioid prescribing practices at Saint Joseph’s University Medical Center (SJUMC). It was our goal to identify factors positively contributing to opioid prescribing to decrease the overall prescriptions of narcotics in an effort to positively impact the opioid epidemic.

## Materials and methods

Seven hundred seventy-four patients who underwent uncomplicated minimally invasive appendectomy and cholecystectomy procedures during a one-year period from May 1, 2017, to April 30, 2018, at SJUMC were evaluated retrospectively after obtaining approval from the institutional review board (IRB). Patients who had complications were removed from the data set as to not skew the information. Demographic variables including sex, surgeon type (hospital vs private), administration of local anesthetic, surgery type, hospital length of stay, and narcotic use in the post-operative anesthesia care unit (PACU) were evaluated. Opioid medications were equilibrated using morphine equivalents (MEQ) [6].

Logistical regression models were created using R version 3.2.4© software (Vienna, Austria: R Foundation for Statistical Computing) to establish which individual variables were most associated with narcotic prescriptions upon discharge. Then, a multivariate logistical regression was performed to find which variables jointly contributed to narcotic prescription. By performing data analysis along with these calculations, we were able to isolate individual variables and determine which factors played a role in patients being discharged with prescriptions for narcotics.

## Results

Our retrospective analysis of 774 patients showed that sex, surgeon type, administration of local anesthetic, surgery type, hospital length of stay, and narcotic use in the PACU were the most statistically significant multivariate factors that correlated discharge with prescription for narcotics (Table 1). Table 1 shows the six previously mentioned significant variables in the univariate regression which affected discharge with narcotics.

**Table 1:** Patient demographics influencing narcotic use with statistical significance. PACU: post-operative anesthesia care unit

Variable		Percentage (%) of patients discharged with opioid scripts	p-Value univariate (receiving narcotic script)	p-Value multivariate (receiving narcotic script)
Gender	Male	51.56	0.00244	0.002093
	Female	63.13		
Physician	Hospital employed attending	54.3	0.0013	1.07E-07
	Private practice attending	64.85		
Surgery type	Laparoscopic appendectomy	57.53	0.00121	0.004645
	Laparoscopic cholecystectomy	57.93		
	Robotic cholecystectomy	93.75		
Amount of local use	<5cc local	59.5	0.0189	0.003271
	5-15cc local	49.23		
	>15cc local	65.9		
Narcotics in PACU	Yes	64.34	0.00147	-
	No	53.04		
Length of stay	Same day	65.63	0.000942	-
	1-4 days	59		
	5-10 days	39.13		
	11+ days	30		

Table 2 demonstrates the breakdown of the demographics of the 774 total patients. Females had a higher likelihood (63% vs 51%) to receive narcotic medications. Additionally, private practice attendings were more likely to prescribe narcotic medications to their patients. Approximately 57-58% of laparoscopic appendectomy and laparoscopic cholecystectomy patients received narcotics upon discharge while 93.7% of the robotic cholecystectomy patients were discharged with narcotics. The amount of local anesthetic use was also statistically significant with 59.5% of patients receiving less than 5 cc’s of anesthetic being discharged with opioid scripts. However, 65.9% of those who received more than 15 cc’s also received opioid scripts upon discharge. Lastly, length of stay in the hospital was associated with 65.6% of patients being discharged on the same day as surgery receiving opioid scripts. This number was reduced as the patient spent more days in the hospital with only 30% of patients receiving scripts when the stay was greater than 10 days.

**Table 2:** Variables categorized into subsets based on total numbers. PACU: post-operative anesthesia care unit

Variable		Total points	Percentage
Gender	Male	256	33%
	Female	518	67%
Physician	Hospital employed attending	407	53%
	Private practice attending	367	47%
Surgery type	Laparoscopic appendectomy	219	28%
	Laparoscopic cholecystectomy	523	68%
	Robotic cholecystectomy	32	4%
Amount of local use	<5cc	110	14%
	>5cc	664	86%
Narcotics given in PACU	Yes	345	45%
	No	429	55%

## Discussion

The epidemic of opioid abuse and opioid overdosing has prompted a rethinking of pain management in surgical patients. In 2019, over 70,500 people died from drug overdose. Additionally, another 10.1 million people misused prescription opioids that same year. A significant of those people were first-time users. It is estimated that approximately 1.6 million people misused prescription pain relievers for the first time [7]. Approximately 8-12% of patients prescribed opioids for chronic pain develop an opioid use disorder [8]. In this retrospective study of 774 patients, our aim was to designate variables that correlated significantly with patients receiving prescriptions for opioid medications upon discharge. It was found that sex, surgeon type, administration of local anesthetic, surgery type, hospital length of stay, and narcotic use in the PACU were all factors that affected this outcome (Table 1).

As mentioned, approximately 63% of females in this study received opioid scripts as opposed to 52% of males. According to Bartley and Fillingim, sex differences in pain showed that women have an increased risk of severe clinical pain as well as chronic pain [9]. However, our study also showed that a higher percentage of private surgeons (64.8%) were more likely to prescribe opioid scripts upon discharge than hospital employed attendings (54.3%). Interestingly, patients who received more local anesthetic tended to be sent home with narcotics. Further, 65.9% of patients who received more than 15 cc’s of local anesthetic received discharge opioid scripts while only 59.5% of patients who received less than 5 cc's did.

All of these variables may be due to the surgeon’s perception of the patient’s future pain requirement and preference of individual surgeons. The lack of standardized pain control regimen post-operative allows for individual surgeon preferences to have a significant impact on opioids over prescription which in turn can have a negative impact on the opioid epidemic.

Robotic cholecystectomies are also more likely to get a narcotic script than laparoscopic appendectomies and laparoscopic cholecystectomies (93.7% vs 57.5% vs 57.9%, respectively) (Table 1). Since most of the robotic cases were performed without residents and by few attendings, it is possible to deduce that this could be private attending preference. In a retrospective study conducted by Betcher et al., patients who underwent robotic hysterectomy vs the traditional procedure had statistically decreased post-operative pain and narcotic use [10]. Our study shows a strong foundation to start decreasing opioid prescriptions in patients who undergo uncomplicated robotic surgeries. This would help alleviate the strain of the opioid epidemic on the community.

When evaluating which patients get narcotics earlier in the PACU, there are a few notable variables. Our study showed statistically that 64% of patients receiving narcotics in the PACU were discharged with opioid scripts. Decreasing narcotic use in the PACU would help reduce the number of patients that are discharged with opioid scripts. In a retrospective study conducted by Chavez et al., using an opioid sparing protocol pre-intervention and post-intervention was effective in managing acute pain in the PACU. It also reduced PACU length of stay and improved patient outcomes [11]. Implementing different types of pain control as well as alternate timings could create avenues in which the use of opioids can be decreased.

A longer post-operative stay was shown less likely result in narcotic prescription on discharge (Table 1). This may be due to a better assessment of pain control requirements in patients whose local anesthetic has worn off and have had the opportunity to declare their true medication requirements. Additionally, different methods of pain control have been implemented in order to decrease opioid use. One example is the enhanced recovery after surgery (ERAS) protocol. This strategy uses non-steroidal anti-inflammatory drugs (NSAIDs), gabapentinoids, and alternative therapies to treat post-operative pain. In a study conducted by Echeverria-Villalobos et al., it was seen that post-operative patients treated with the ERAS protocol had decreased administrations of opioid medications [12]. Another strategy that was investigated by Mulita et al. studied the use of intravenous (IV) acetaminophen with intramuscular (IM) pethidine, acetaminophen with IV parecoxib, and acetaminophen monotherapy. It was shown that combinations of IV acetaminophen with either IM pethidine or IV parecoxib were superior to IV acetaminophen monotherapy in patients undergoing open inguinal hernia repair and laparoscopic cholecystectomy [13,14]. This drug regimen provides another potentially efficacious modality to treat post-operative pain.

This study is limited by retrospective analysis and therefore we can only draw conclusions from a non-controlled data set. At the time of creation of this study, there was no set protocol for pain control medications or dosing. Currently, in a study by Chou et al., it is recommended that operative pain management should begin in the pre-operative period. This should be carried out through the post-operative period and should be tailored individually to each patient [15]. Our data stem entirely from the previously established practices and habits, with perhaps antiquated motives such as to help get patients home as soon as possible. Additionally, with there being one hospital system, there is the limitation of selection bias and patient population.

## Conclusions

The link between the prescription of opioids and eventual opioid addiction has been clearly demonstrated. The lack of protocolized peri-operative pain management strategies has resulted in a wide variation in prescribing practices. Hospital systems and teaching institutions should use their influence over surgeons and residents to shape evidence-based prescribing practices to limit opioid prescriptions. This can be achieved by standardizing the use of NSAIDS and local anesthetics to limit post-operative pain. Patients should also be monitored to accurately assess their pain medication requirements. These protocols and best practices may be best implemented in a multidisciplinary effort to create order sets and quality metrics to ensure enforcement.
